# High-yield Production of Granulocyte-macrophage Colony-stimulating Factor in *E. coli* BL21 (DE3) By an Auto-induction Strategy

**Published:** 2019

**Authors:** Raziyeh Malekian, Ali Jahanian-Najafabadi, Fatemeh Moazen, Reza Ghavimi, Elmira Mohammadi, Vajihe Akbari

**Affiliations:** *Department of Pharmaceutical Biotechnology, Isfahan Pharmaceutical Research Center, Faculty of Pharmacy, Isfahan University of Medical Sciences, Isfahan, Iran.*

**Keywords:** Granulocyte-macrophage colony-stimulating factor, Auto-induction, Inclusion bodies, *Escherichia coli*, Overexpression.

## Abstract

A novel strategy to increase protein expression yield is unintended induction of expression in complex media, called auto-induction. This method can be used to express proteins under control of the *lac* promoter without any need to monitor bacterial growth pattern, and addition of specific expression inducers such as Isopropyl β-D-1-thiogalactopyranoside (IPTG) at proper time. In the present study, a codon optimized gene encoding granulocyte-macrophage colony stimulating factor (GM-CSF) was cloned and over-expressed in *Escherichia coli* BL21 (DE3) using both conventional inducer-based and auto-induction approaches in a shake flask scale and the yield of GM-CSF expression and biomass production was identified. Results showed higher biomass production and expression yield for GM-CSF in case of auto-induction comparing with IPTG-induction. The auto-induction approach was also performed in a fed batch fermentation process in a 2-L bioreactor scale. The feeding strategy yielded an amount of 300 mg/L GM-CSF within 20 h of induction. However, most of the over-expressed GM-CSF was produced as inclusion bodies and following purification and refolding, a final yield of 90 mg/L was achieved. These results suggest that auto-induction approach can be effectively applied in fed-batch fermentation for the large scale production of GM-CSF; however, further optimization of purification process is obligatory to increase the purification yield.

## Introduction

Granulocyte-macrophage colony-stimulating factor (GM-CSF) is a member of colony-stimulating factors which regulates proliferation, differentiation, and survival of hematopoietic cells including mature neutrophils, macrophages, and dendritic cells (1). This cytokine is secreted by a large variety of cells including lymphocytes (B and T), macrophages, fibroblasts, and endothelial cells in response to informatory stimuli like cytokines and microbial products (2). GM-CSF has been approved for treatment of neutropenia due to myelosuppressive chemotherapy or bone marrow transplantation, peripheral blood stem cell mobilization, graft failure and induction therapy for acute myelogenous leukemia (AML) (3). Today, two GM-CSF analogs are available for clinical applications; one of them is a glycosylated protein derived from yeast expression system using *Saccharomyces cerevisiae *(Sargramostim™), while the other one is a non-glycosylated protein derived from *Escherichia coli* expression system (Molgramostim™) (4, 5). There is no significant difference in efficacy and side effects between yeast derived and *E. coli* derived GM-CSF. However, non-glycosylated GM-CSF (produced in *E. coli*) shows more *in-vitro* biological activity and less half-life compared with glycosylated protein (6).

*E. coli* is one of the most popular expression platforms for production of heterologous proteins (7). The advantages of using *E. coli* expression system for production of recombinant proteins include well-characterized genetics and biochemical properties, easy manipulation and scale up, and low cost of high cell density growth for expression of higher levels of heterologous proteins (8). Many attempts have been made to improve productivity and effectiveness of this workhorse microorganism (9); however, there is no guarantee that a heterologous gene product will be expressed in *E. coli* at high levels and in a full biologically active form (10). For example, there have been some reports on expression of GM-CSF in *E. coli*, but the expression level was usually low due to poor stability of the protein expressed in the cytoplasm, and also the post induction cell lysis because of the toxicity of the recombinant protein (11). 

A novel strategy to increase protein expression yield is auto-induction which can be used to express proteins under the control of the *lac* promoter without adding IPTG or other external inducers (12). Its principle is based on using auto-induction media containing multiple carbon sources including glucose, glycerol, and lactose (13). Glucose prevents expression leakage by catabolic repression and allows cell growth to saturation without induction. Upon depletion of glucose, lactose activates the *lac* promoter to induce the protein expression (14). Glycerol and lactose provide sustained growth during the induction phase (13). Auto-induction cultures are simply inoculated into a suitable auto-inducing medium and grown to saturation without the need to monitor and add inducer at the appropriate cell density, which is much more convenient, effective, and economical than conventional IPTG induction (15). This novel induction strategy is particularly suitable for high level expression at high density culture and for the expression of toxic proteins (13, 16). Long *et al.* reported that auto-induction strategy can significantly improve expression of recombinant human tissue plasminogen activator (tPA), a protease with potential toxicity for host bacteria (17). However, this strategy has not yet been reported for *E. coli* expression of GM-CSF in shake flask or bioreactor.

In the current study, a codon optimized gene encoding GM-CSF was cloned and highly expressed in *E. coli* BL21 (DE3) using both conventional IPTG induction-based and auto-induction approach in a shake flask scale. In addition, the auto-induction approach was performed in fed batch fermentation in a 2-L bioreactor scale. Furthermore, here different aspects regarding purification and refolding procedures following auto induction of the recombinant GM-CSF are reported and discussed.

## Experimental

*Bacterial strain and plasmid*

The amino acid sequence of human GM-CSF was used to design a codon optimized DNA sequence (*rGM-CSF*) for its expression in *E. coli*. The sequence also included NcoI (5′) and XhoI (3′) restriction sites, an amino-terminal 6xHis-tag and a stop codon. The fragment was synthesized by Biomatik Company (Canada) and obtained in pET28a expression plasmid (Novagen, USA). Finally, *E. coli *BL21 (DE3) was transformed with the recombinant pET28-GM-CSF and used for subsequent expression experiments.

*Auto-induction medium*

Auto-induction expression was performed in ZYP-5052 medium prepared based on Studier‘s method for auto-induction with some modification (13). The ZYP-5052 medium consisted of the following components (in g L^-1^): 0.5 glucose, 2.0 lactose, 5.0 glycerol, 10.0 tryptone, 5.0 yeast extract, 7.1 Na_2_HPO_4_, 6.8 KH_2_PO_4_, 3.3 (NH_4_)_2_SO_4_, 0.12 MgSO_4, _and 1X metal solution. The metal stock solution (1,000X) included 50 mM FeCl_3_, 20 mM CaCl_2_, 10 mM each of MnCl_2_ and ZnSO_4_, and 2 mM each of CoCl_2_, CuCl_2_, NiCl_2_, Na_2_MoO_4_, and H_3_BO_3_ (Table 1). Kanamycin (Sigma, Germany) was used at a concentration of 100 µg mL^-1^ (higher concentration of kanamycin is required for selection in the phosphate rich medium (*e.g.,* ZYP-5052) compared with other media; high phosphate decrease the sensitivity to kanamycin (13). 

*General expression of rGM-CSF*

A single recombinant colony was transferred to 5 mL of Luria–Bertani (LB) broth containing 25 µg/mL kanamycin, and the culture was incubated at 37 °C and 180 rpm for 16 h. On the following day, this culture was added to 50 mL fresh LB medium in 150 mL shaker flask and the cells were incubated at 37 °C until they reached the exponential phase (an OD_600_ of 0.4-0.6). Then, expression of the protein was induced by addition of 1 mM IPTG at 37 °C for 3 h.

*Small scale auto-induction*

A single recombinant colony was transferred to 5 mL of LB broth supplemented with 1% glucose and 25 µg/mL kanamycin, and was incubated at 37 °C and 180 rpm for 8 h. This culture was used to inoculate 50 mL of ZYP-5052 medium at a ratio of 1:100 and incubated overnight at 37 °C.

*Large scale auto-induction*

Bioreactor scale cultivation and production was performed in a 2-L stirred-tank bioreactor (BioTron, Korea) at 37 °C for 25 h. The pre-inoculum culture was transferred to a 250 mL shaker flask containing 50 mL LB broth supplemented with 1% glucose and shaken at 37 °C and 180 rpm overnight. This culture was inoculated (1:1000) into the bioreactor containing 1250 mL of ZYP-5052 medium under sterile conditions. During the entire process, the dissolved oxygen level (DO) was maintained at approximately 30% air saturation by automatically adjusting the agitation speed and air flow rate. Antifoam (Propylene glycol) was added manually when necessary. The supplementary medium (112.5 mL), containing glycerol and lactose as carbon sources and inducer, (NH_4_)_2_SO_4_ as a nitrogen source, and Na_2_HPO_4_ ,KH_2_PO_4_ as buffering agents, was added after 7 h as a pulse.

**Table 1:** Composition of media used in this study

**Ingredients**	**Auto-induction**		**Conventional**	
	**Batch**	**Pulse**	**LB**	**LB with glucose**
Yeast extract (g L-1)	5	-	5	5
Tryptone (g L-1)	10	-	10	10
NaCl (g L-1)	-	-	10	10
Glucose (g L-1)	0.5	-	-	10
Lactose (g L-1)	2	66.7	-	-
Glycerol (g L-1)	5	162.2	-	-
MgSO4 (mM)	1	-	-	-
Na2HPO4 (g L ) -1	7.1	15.8	-	-
KH2PO4 (g L ) -1	6.8	15.1	-	-
(NH4)2SO4(g L ) -1	3.3	7.3	-	-
1000x trace metal solution	1x	-	-	-
Kanamycin (mg L-1)	100	-	25	25

**Table 2:** Summary of the yields of rGM-CSF expressed by auto-induction method including data of various steps of purification and refolding

**Procedure**	**rGM-CSF (mg)**	**Purity (%)**	**Yield (%)**
Whole cell lysate^a^	15	25	100
Soluble protein	1.5	1.5	10
Insoluble protein	13.5	24	90
Denaturing NI-NTA purification	6	90	44.4
Refolding	4.5	92	75

a From pellet obtained from 50 mL of cell culture.

**Figure 1:**
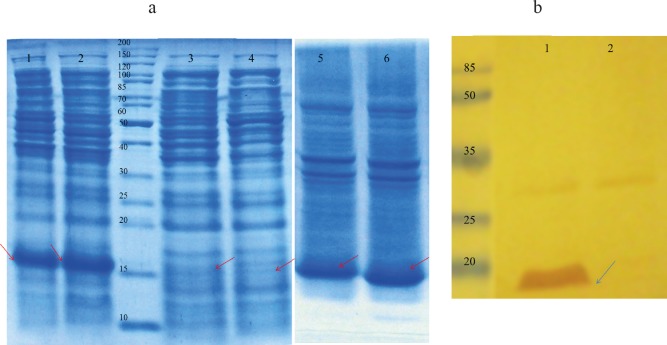
(a) Proteins were separated on a 15 % SDS-PAGE and visualized by Coomassie brilliant blue R250 staining. Lane 1: total protein, after induction with 1 mM IPTG for 3 h at 37 °C IPTG-induction; Lane 2: total protein after auto-induction for 8 h at 37 °C; Lane 3: soluble cytoplasmic fraction following auto-induction for 7 h at 37 °C; Lane 4: soluble cytoplasmic fraction after induction with 1 mM IPTG for 3 h at 37 °C; Lane 5: insoluble cytoplasmic fraction after induction with 1 mM IPTG for 3 h at 37 °C; Lane 6: insoluble cytoplasmic fraction following auto-induction for 7 h 37 °C. (b) Western blot analysis with anti-His antibody. Total protein extracted from *E. coli *BL21 (DE3) containing pET28a (*rGM-CSF*) after induction (lane 1) and before induction as negative control (lane 2). rGM-CSF (16 kDa) is denoted by arrows

**Figure 2:**
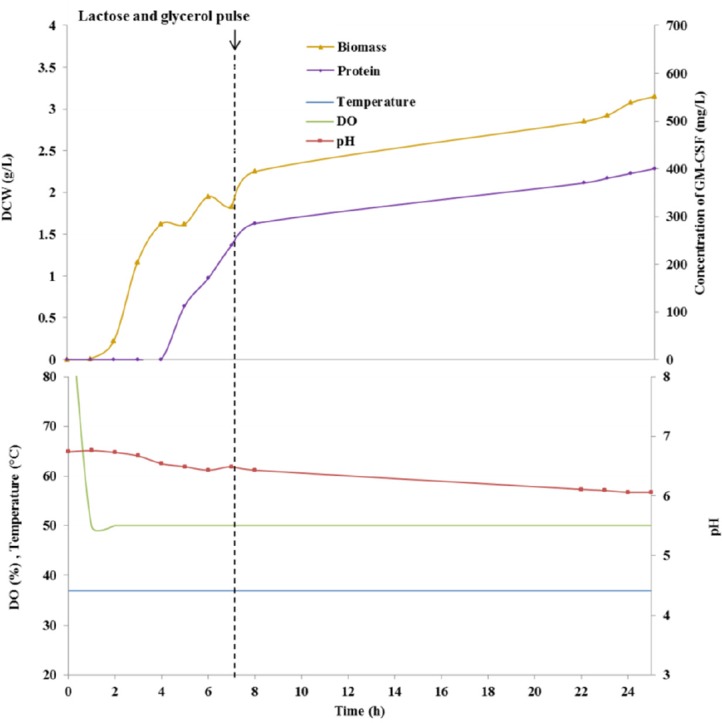
Fed-batch culture course in a 2-L fermenter using auto-induction medium

**Figure 3:**
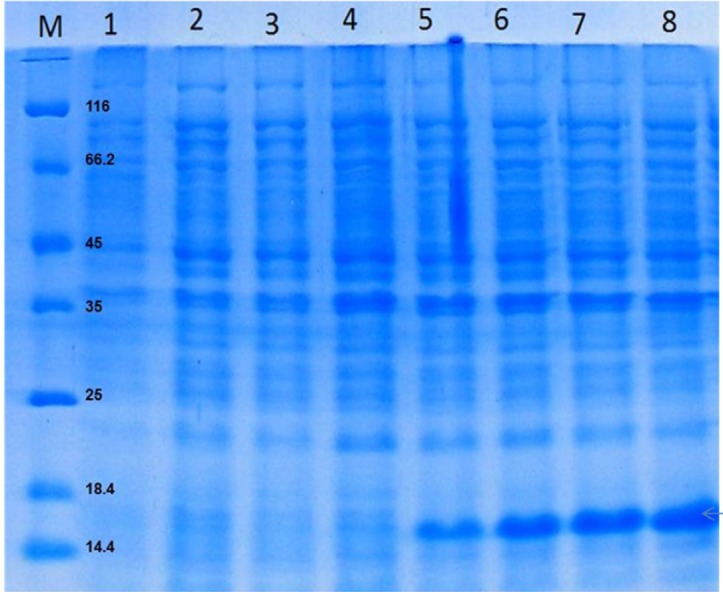
Proteins were separated on a 15% SDS-PAGE gel and visualized by Coomassie brilliant blue R250 staining. Total protein of auto-induction culture in a 2-L bioreactor, after 1, 2, 3, 4, 5, 6, 7 and 8 h (lanes 1-8) at 37 °C. rGM-CSF (16 kDa) is denoted by arrows

**Figure 4:**
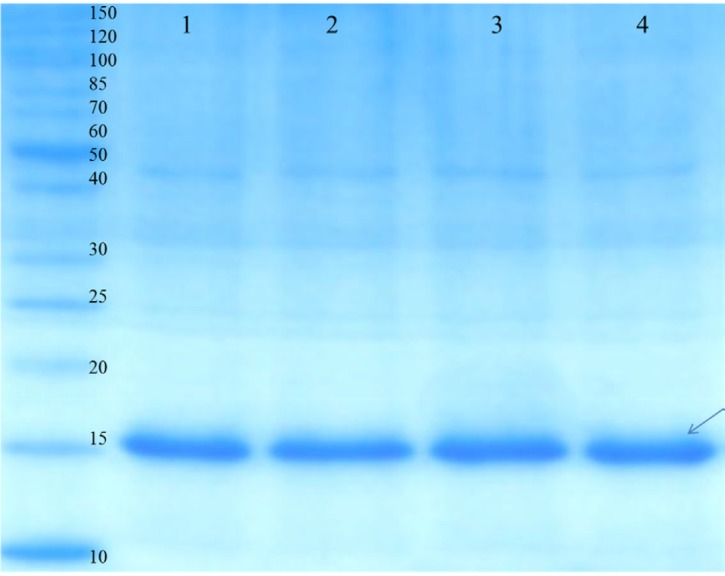
SDS-PAGE analysis of the purified rGM-CSF. Proteins were separated on a 15% SDS-PAGE gel and visualized by Coomassie brilliant blue R250 staining. Lane 1, 2, 3 and 4 corresponds to elutions 1-4, respectively. rGM-CSF (16 kDa) is denoted by arrows

*Purification*

At the final expression time, the cells were harvested by centrifugation at 7,500×*g* for 10 min and the pellet was resuspended in cold lysis buffer (50 mM Tris, 1 mM DTT and 100 mM NaCl, pH 8.0), and subsequently disrupted through sonication (Hielscher, Germany) in an ice-bath. This lysate was incubated in the presence of 10 U/mL benzonase (Sigma, USA) for 15 min at 25 °C to eliminate DNA and RNA. The soluble and insoluble fractions of the cell lysate were separated by centrifugation at 7,500×g for 10 min. The insoluble fraction was solubilized with guanidinium lysis buffer and centrifuged at 7,500×g for 10 min to remove the insoluble debris. The obtained supernatant was loaded onto a nickel affinity column (Ni-NTA Resin, Invitrogen, USA) and the recombinant GM-CSF was purified as described by the manufacturer (18). Briefly, the column was equilibrated with denaturing binding buffer (8 M urea, 20 mM sodium phosphate, 500 mM NaCl, pH 7.8). Then, the column was washed two times with denaturing wash buffer 1 (8 M urea, 20 mM sodium phosphate, 500 mM NaCl, pH 6.0) followed by three washes with denaturing wash buffer 2 (8 M urea, 20 mM sodium phosphate, 500 mM NaCl, pH 5.3). Finally, the recombinant GM-CSF was eluted in 1 mL aliquots using denaturing elution buffer (8 M urea, 20 mM sodium phosphate, 500 mM NaCl, pH 4).

*Refolding*

The purified rGM-CSF (5 mL) was dialyzed against 500 mL of refolding buffer 1 (50 mM Tris, 2 M Urea, pH 8) at 4 °C overnight to slowly remove urea. The next day, the protein solution was dialyzed overnight at 4 °C against 500 mL of refolding buffer 2 (50 mM Tris, 0.5 M urea, 0.1 M arginine, 1 mM reduced glutathione, 0.1 mM oxidized glutathione, pH 8). Finally, the dialysis buffer was replaced with refolding buffer 3 (50 mM Tris, 250 mM NaCl, 50 mM arginine, 1 mM reduced glutathione, 0.1 mM oxidized glutathione, pH 8) and dialyzed overnight at 4 °C. 

*Analytical methods*

During the bioreactor cultivations, the samples were taken every hour for cellular growth and protein analysis. The cell growth was monitored using measurements of the optical density (OD) of culture medium at 600 nm. In this regard, the samples were diluted between 1:1 and 1:20 in 0.9% NaCl solution. 

For determination of dry cell weight (DCW), 10 mL of the samples were harvested and centrifuged at 4 °C and 4,700 rpm. Afterwards, the cell pellets were washed with 10 mL of 0.9% NaCl solution and centrifuged again. The supernatants were removed and the cell pellets were dried overnight at 80 °C before being weighed. Protein expression was evaluated by 15% sodium dodecyl sulfate polyacrylamide gel electrophoresis (SDS-PAGE) and the protein production rate was estimated by densitometry analysis of acrylamide gels using TL120 software (Nonlinear Inc, Durham NC, USA) (18). Bovine serum albumin samples with known concentration (0.0312, 0.0625, 0.125, 0.25 and 0.5 mg/mL) were used as standards to estimate rGM-CSF concentration on gels. The concentration of rGM-CSF after purification and refolding was determined according to the Bradford method.

## Results

*Protein expression in shake flask*

The *rGM-CSF* coding sequence was synthesized on the basis of codon optimized nucleotide sequences corresponding to the amino acid sequence of Molgramostim^TM^ and the synthesized gene was obtained as an expression frame in the pET28a plasmid (*pET28-GM-CSF*). *E. coli *BL21 (DE3) harboring the *pET28-GM-CSF* plasmid was used for over-expression of the rGM-CSF using both conventional IPTG induction and auto-induction approaches in shake flask scale, and the expression was analyzed by SDS–PAGE. As it is shown by Figure 1a, both approaches led to over-expression of rGM-CSF, but the auto-induction produced approximately 33% more rGM-CSF compared to the IPTG induced expression. While the high level of rGM-CSF expression (approximately 25% of total cellular protein content) could be achieved, most of the over-expressed protein was in the insoluble fraction. 

The identity of the over-expressed protein was verified by Western blotting using anti-6X His-tag antibody. Western blot analysis indicated that the expressed rGM-CSF was a His-tagged fusion protein with a molecular weight of 16 kDa (Figure 1b).

*Auto-induction of rGM-CSF in a 2-L bioreactor*

The profiles of auto-induced culture of *E. coli* BL21 (DE3) harboring pET28a (*rGM-CSF*) in a 2-L bioreactor was presented in Figure 2. The cellular growth and the levels of biomass were monitored based on measurement of DCW and OD_600_. OD_600_ of 1 corresponded to approximately 0.395 g of DCW per liter culture of *E. coli* BL21 (DE3). The cellular growth was illustrated with a diauxic (two phase) growth curve. First, an exponential cell growth was observed, as the bacterium consuming glucose has the higher maximum specific growth rate. The initial carbon source was exhausted after 5 h of cultivation and following a lag phase, cellular growth continued with a lower growth rate because of the application of lactose as the main carbon source. In the auto-induction approach, the cellular concentration reached an OD_600_ of 8 within 25 h of cultivation.

As shown in Figure 3, protein expression was induced 5 h after cultivation. It must be noted that under auto-induction condition, biomass formation continued even following expression induction because the cells were not exposed to a prolonged induction regimen. At 7 h of cultivation, a pulse feeding of a solution containing glycerol and lactose as carbon sources and inducer, (NH_4_)_2_SO_4_ as a nitrogen source, and Na_2_HPO_4_, KH_2_PO_4_ as buffering agents were added, in order to avoid reduction of protein synthesis rate as a result of insufficient carbon source or inducer. The use of this feeding strategy yielded a yield of 300 mg/L rGM-CSF within 20 h of induction, corresponding to a protein-specific production rate of 95 mg_protein_/g _DCW_.

*Purification and refoding of GM-CSF*

Purification of rGM-CSF was performed using Ni-NTA affinity chromatography under denaturing condition which led to about 90% purity (Figure 4), and overall yield of rGM-CSF was 38 mg/g DCW. While rGM-CSF was mainly expressed as inclusion bodies (insoluble proteins), most of it was purified by Ni-NTA affinity chromatography and the yield of this procedure was 44.4% (Table 2). The denatured rGM-CSF was dialyzed against buffers containing arginine, and reduced and oxidized glutathione to slowly reduce the urea concentration of the protein solution which resulted in high refolding yield of 75% at high protein concentrations (Table 2).

## Discussion

Considering the wide range of GM-CSF therapeutic applications, various strategies for production of proper amounts of this protein with reasonable costs have been considered. Here, we reported high-yield production of rGM-CSF in *E. coli* by an auto-induction strategy for the first time. To the best of our knowledge, this is the first study that described the scale-up of GM-CSF auto-induction from shake flask to fed-batch bioreactor scale. 

The yield of GM-CSF production reported by most of the published approaches is diverse and often contradictory. Over-expression of GM-CSF in cytoplasm of *E. coli* has been reported to be very difficult because bacterial cells stopped growing and eventually lysed upon induction due to toxicity of the recombinant protein (11). Bacterial expression of human GM-CSF was firstly described by Burgess and coworkers using a temperature inducible plasmid in *E. coil *(19). They reported expression of GM-CSF as inclusion bodies which corresponded to 8% of total cell protein (19). Oloomi *et al*. observed that GM-CSF accumulated as insoluble inclusion bodies with a yield of 1 mg/L of bacterial culture (20). Another group reported that cytoplasmic expression of GM-CSF using IPTG induction yielded 0.4 mg of bioactive protein per liter of bacterial culture (21). Furthermore, supplementation with a tRNA for a rare codon (AGA/AGG), which codes for arginine, resulted in 3-4-fold enhancement of GM-CSF expression (22). Another study reported that gene optimization of the first sixteen N-terminal amino acids of GM-CSF led to significant increase in its expression level (23). There are also some successful reports on production of rGM-CSF as fusion protein. For example, rGM-CSF was expressed as an intein fusion protein in *E. coli* BL21 (DE3), and *E. coli* GJ1158, and the estimated production yield following intein-mediated purification reported to be 7 mg/L and 20 mg/L, respectively (24). Alternatively, we used auto-induction strategy for successful over-expression of codon optimized rGM-CSF in *E. coli* cytoplasm which yielded 90 mg/L of purified protein. Auto-induction as a convenient, economic, and easy automatized method can be applied for high production of GM-CSF and other recombinant proteins. 

Grossman *et al*. observed that production of recombinant proteins using T7 expression system without application of inducer significantly increased as cells grown in complex medium enter the stationary phase (25). However, here we observed that auto-induction started when cells were at the late exponential growth phases (4-5 h). This indicated that auto-induction could start within a few hours and did not necessarily occur upon approach to saturation and high density culture which usually took long time.

Scale-up of the auto-induction procedure from shake flasks to bioreactors is not always straightforward, because of various factors affecting cell growth and protein expression, such as the rate of aeration and pH, which are not well monitored and controlled in shake flasks (26). In this study we used ZYP-5052 medium (50 mM phosphate) which prevents pH decrease to a level that stops cell growth during fermentation. High buffering capacity of this media led to small variation in pH and the final saturated cultures had a pH greater than 6.0. Based on oxygen saturation, *E. coli* cells prefer utilization of glycerol or lactose as a carbon source (27). The level of oxygen saturation in auto-induction culture can affect the yield of biomass and protein production (13). Ukkonen *et al*. used ZYM-5052 auto-induction medium (lower phosphate salts concentration and lower buffering capacity compared with ZYP-5052 medium) and reported higher cell density and lower expression yield for high oxygenation conditions (Maximum oxygen transfer rate (OTR_max_)∼120 mmol L^−1^ h^−1^) compared to moderately aerated conditions (OTR_max_∼30 mmol L^−1^ h^−1^) (28). Under highly aerated conditions, glycerol is mainly utilized as a carbon source and prevents lactose consumption. Also, cultivation using a high lactose medium increases lactose induction and utilization. Auto-induction medium applied in this study, ZYP-5052, with 0.5 g/L glucose, 2 g/L lactose and 5 g/L glycerol necessitated well-controlled rate of aeration (13). Therefore, throughout the bioreactor cultivation, DO was maintained at approximately 30% air saturation to provide sufficient oxygen leading higher cell density culture compared to shake flask (an OD_600_ of 8 compared with an OD_600_ of 2, respectively). However, Mayer and coworkers (29) performed cultivation and production of recombinant PDI-A protein in a highly aerated condition (80%) using a low lactose auto-induction medium with an enzymatic glucose releasing system, and without glycerol. They obtained high cell (OD_600_ = 25) and product (1.3 g/L) yields without oxygen limitation (29). They concluded that unlike glycerol-based auto-induction systems, this system, tolerates different oxygen saturation conditions (29).

## Conclusion

In the present study, we reported an effective strategy for over-expression of rGM-CSF in cytoplasm of *E. coli*. The over-expressed protein mainly aggregated as inclusion bodies which can be purified using one-step affinity chromatography under denaturing conditions and subsequently refolded *in-vitro* by slow dialysis against buffers containing arginine and a combination of reduced and oxidized thiols. Moreover, we successfully scaled up auto-induction of GM-CSF from shake flask to fed-batch bioreactor. A pulse feeding of lactose and glycerol was used to provide adequate inducer and carbon source during 25 h of cultivation. Considering different therapeutic and research applications of GM-CSF, the auto-induction approach has the potential to be used for economical production of this protein in large amounts. Furthermore, the efficiency of the purification and refolding procedures are going to be evaluated by undergoing studies on the structure and biological activity of the purified rGM-CSF.
